# Specializing

**Published:** 1881-01

**Authors:** 


### Editorial.

### SPECIALIZING,

Dr. Geo. A. Mills, D. D. S., of Brooklyn, N. Y., who is well known to the dentists of this country, and to many in other countries as well, has taken up and will make a specialty of the treatment of diseases of the gums, particularly those affections often denominated "Riggs Disease," "Pyorrhoea Alveolaris" loosened teeth and atrophid conditions of the surroundings. This is well. When any one attains extraordinary ability in any particular direction, it is not only proper, but desirable that he should use his knowledge and skill for the benefit of all who need, and can make it available. In larger cities such specializing can with very great advantage be adopted and followed. There are a few who give exclusive attention to Prosthetic Dentistry, and the degree of efficiency and perfection to which they have attained in this branch, is an unanswerable argument in favor of such a course.

For the most part, the best operators upon the natural teeth, are those who give almost or quite exclusive attention to that branch of practice.

It is proper and desirable that every one should select that for which he is best adapted, and pursue it fully as circumstances will admit; then and then only will the greatest progress and highest results be attained.

